# Influence of the COVID-19 Pandemic on the Subjective Life Satisfaction of South Korean Adults: Bayesian Nomogram Approach

**DOI:** 10.3390/diagnostics12030761

**Published:** 2022-03-21

**Authors:** Haewon Byeon

**Affiliations:** 1 Department of Medical Big Data, College of AI Convergence, Inje University, Gimhae 50834, Korea; bhwpuma@naver.com; Tel.: +82-10-7404-6969; 2 Department of Digital Anti-Aging Healthcare (BK-21), Graduate School, Inje University, Gimhae 50834, Korea

**Keywords:** life satisfaction, COVID-19 pandemic, Bayesian nomogram, multiple risk factors, community-dwelling adults

## Abstract

To understand the changes in the lives of adults living in local communities due to the COVID-19 pandemic, it is necessary to identify subjective life satisfaction and to understand key factors affecting life satisfaction. This study identified the effect on life satisfaction of COVID-19 using epidemiological data representing adults in South Korean communities and developed a model for predicting the factors adversely affecting life satisfaction by applying a Bayesian nomogram. The subjects of this study were 227,808 adults who were 19 years old or older. Life satisfaction was measured in units of 10 points from 0 to 100: a score of 30 or less corresponding to −1 standard deviations was reclassified as dissatisfied, and a score of 40 or more was reclassified as satisfied. The nomogram developed in this study showed that “females who were between 30 and 39 years old, living in urban areas, with fewer meetings and sleeping hours, concerned about infection for themselves and the weak in the family due to the COVID-19 pandemic, concerned about death, with a mean household monthly income of KRW 3–5 million, who were non-smokers, with poor subjective health, and an education level of college graduation or above” would have a 66% chance of life dissatisfaction due to the COVID-19 pandemic. The results of this study suggest that the government needs not only to provide economic support but also to support education on infectious diseases and customized psychological counseling programs for those at high risk of life dissatisfaction after the COVID-19 pandemic.

## 1. Introduction

As social distancing minimizing human contact has become a common lifestyle due to the prolonged global COVID-19 pandemic, the activities of daily life, such as education and leisure activities, have been changed considerably. As the lockdown due to COVID-19 continues, face-to-face meetings with other people have decreased, and depression and anxiety have been amplified due to physical and mental health vulnerabilities caused by the uncertainty regarding infection [1,2]. It has been reported that the lockdown experience and psychological difficulties due to COVID-19 have aggravated individual stress [3]. Fujiwara et al. (2022) [4] showed that people’s work–life balance was worse after the COVID-19 pandemic than before the COVID-19 pandemic, and chronic fatigue and depression also increased.

The experience of such a disaster can adversely affect society in various ways, and the resulting changes in daily life and emotional problems are likely to continue even after the pandemic is over. For example, many people and patients experienced anxiety and fear due to the acute severe respiratory syndrome (SARS) outbreak in Hong Kong in 2003 and that of Middle East respiratory syndrome (MERS) in 2015, and 70.8% of confirmed patients experienced psychiatric problems such as depression, insomnia, and changes in their daily lives, even after the epidemic was over [5]. It is highly likely that social changes such as the expansion of a contactless culture that avoids face-to-face contact between people and the increase in telecommuting will spread even after the COVID-19 pandemic is over, considering the current prolonged COVID-19 pandemic situation. Therefore, to understand the changes in the lives of adults living in local communities, due to the COVID-19 pandemic, it is necessary to identify subjective life satisfaction and to understand key factors affecting life satisfaction.

Life satisfaction is defined as subjective well-being. It is similar to the concept of happiness and refers to the overall quality of an individual’s life. In other words, life satisfaction is a subjective perception or emotion that best reflects the overall current situation of an individual’s life [6]. Previous studies [7,8,9] that evaluated life satisfaction in communities have three limitations: (1) most of them were conducted before the COVID-19 pandemic, (2) they mostly focused on identifying individual factors affecting life satisfaction using regression analysis, and (3) it was difficult for them to measure life satisfaction in detail because they measured subjective life satisfaction on a three-point scale (good, average, or bad) or a five-point scale (very good, good, average, bad, or very bad).

As of February 2022, it has been only 23 months since the COVID-19 pandemic occurred, and only a handful of epidemiological studies have evaluated the life satisfaction of communities due to COVID-19. In particular, it is necessary to develop a predictive model considering multiple risk factors to predict groups with lowered life satisfaction, because life satisfaction is influenced by the interaction of various factors rather than by only a single factor.

Over the past decade, several studies [10,11,12] have used the Bayesian nomogram to identify those at high risk of a target variable such as a disease, considering multiple risk factors. The nomogram is a graph that visualizes the prediction function derived from a Bayesian algorithm or a logistic algorithm in two dimensions, to help healthcare workers understand the information more easily. It has mainly been used in the medical field, such as for the diagnosis of cancer [13] or dementia [14]. In particular, since the Bayesian nomogram is used for predicting the probability of disease occurrence due to multiple risk factors by summing the individual risk factors included in the predictive model [11], it can be effectively applied to predict life satisfaction after the COVID-19 pandemic. This study identified how COVID-19 affected life satisfaction using epidemiological data (*n* = 227,808) representing adults in South Korean communities and developed a model for predicting the factors adversely affecting life satisfaction by applying a Bayesian nomogram.

## 2. Method

### 2.1. Subjects

The data source for this study was the Community Health Survey 2020. The Community Health Survey was approved by the Research Ethics Review Committee of the Centers for Disease Control and Prevention (No. 2016-10-01-P-A). The 2020 Korean Community Health Survey, conducted by the Korea Centers for Disease Control and Prevention, collected data from 17 cities and provinces across South Korea. The subjects of the survey were adults (≥19 years) living in the local community at the time of the survey, and the samples were extracted using the proportional systematic sampling method [15]. The 2020 Korean Community Health Survey collected data from 16 August to 31 October 2020. Data were collected by a trained surveyor who visited the sample households in person and conducted an interview using a laptop equipped with a questionnaire program. Please refer to Kang et al. (2015) [15] for further details of the data collection and other procedures of the Korean Community Health Survey. The subjects of this study were 227,808 adults who were 19 years old or older.

### 2.2. Measurement

The target variables were defined as the subjective life satisfaction during the COVID-19 pandemic. Life satisfaction was measured in units of 10 points from 0 to 100; a score of 30 or less corresponding to -1 standard deviations was reclassified as dissatisfied, and a score of 40 or more was reclassified as satisfied.

The explanatory variables included residential area type (urban or rural), age (19–29, 30–39, 40–49, 50–59, and 60+ years), gender, education level (elementary school graduation or below, middle school graduation, high school graduation, or college graduation or above), mean monthly household income (less than KRW 1 million, KRW 1 to 3 million, KRW 3 to 5 million, or KRW 5 million or more), smoking (non-smoker, past smoker, or current smoker), binge-drinking (binge-drinking was defined as a case of drinking more than seven cups for men and more than five cups for women and drinking more than twice a week over the past year; yes or no), regular exercise (whether the subject did moderate-intensity exercise for at least 30 min per day for at least 5 days per week during the past week; yes or no), subjective health level (good, average, or bad), concerns about COVID-19 infection (concerned, indifferent, or not concerned), fear of death due to COVID-19 infection, concerns about criticism from others due to COVID-19 infection, concerns about family’s COVID-19 infection (e.g., older adults and children), concerns about economic damage (e.g., unemployment) due to COVID-19, changes in sleeping hours after the COVID-19 pandemic (increased, similar, or decreased), and number of meetings with friends or neighbors after the outbreak of COVID-19.

### 2.3. Bayesian Nomogram

**Figure 1:**
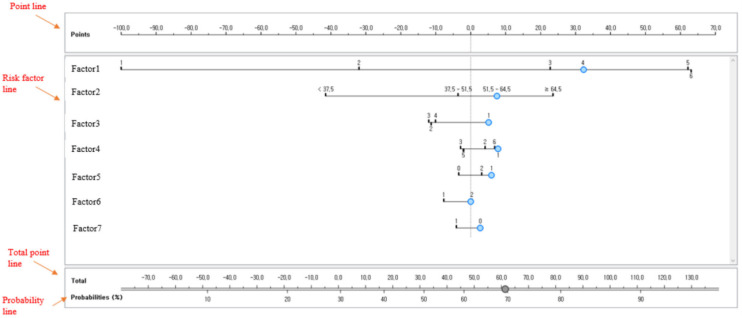
Example of a Bayesian nomogram [11].

Bayesian nomograms were analyzed using Python 3.9.2. The prediction model for the subjective life satisfaction of South Korean adults under the COVID-19 pandemic was developed using a Bayesian nomogram. A Bayesian nomogram consists of 4 types of lines such as a point line, a number of risk factor lines, the total point line, and the probability line. The probability line is the final sum of the nomogram scores calculated using multiple risk factors. Figure 1 presents an example of a Bayesian nomogram.

The development process of the Bayesian nomogram is as follows [11]. First, a point is calculated for each risk factor, where the attribute value is specified as $a_{ij}$, and the value of $LR\left(a_{ij}\right)$ is calculated by Equation (1):

(1) $$LR\left(a_{ij}\right)=\frac{P(a_{ij}|c)}{P(a_{ij}|\bar{c})}=\frac{posterior\;\;odds}{prior\;odds}\;\;\;\;\;\;$$

where, i=1,⋯, m is the number of attributes and $j=1,\;\cdots ,\;n_{i}$ indicates the number of attribute categories. These are calculated by using the point for each risk factor $\left(point_{ij}\right)$ $logLR\left(a_{ij}\right)$ as shown in Equation (2).

(2) $$point_{ij}=\frac{logLR\left(a_{ij}\right)}{max_{ij}\left|logLR\left(a_{ij}\right)\right|}\times 100\;\;\;\;\;\;\;\;\;\;$$

The value of the point assigned to each risk factor ranges from −100 to 100. The risk factor with the largest absolute value of the estimated log-likelihood ratio (e.g., 100 points) was identified as the risk factor with the greatest influence. The scores of the remaining risk factors were calculated by dividing the log-likelihood ratio of the factor by the absolute log-likelihood ratio of the factor with the greatest influence and then multiplying by 100.

This study used 10-fold validation to validate the predictive performance of the Bayesian nomogram. The performance of the Bayesian nomogram was evaluated using F1 score, precision, recall, calibration plot, and general accuracy.

## 3. Results

### 3.1. General Characteristics of Subjects

The general characteristics of all subjects (227,808 people) were analyzed. The mean age of the subjects was 54.4 years (standard deviation = 17.7). Among the subjects, the proportions of those who were 60 years or older (46.6%), female (54.6%), urban dwellers (56.4%), educated to college graduation level or above (37.8%), in households with a mean monthly income of KRW 1 to 3 million (31.8%), non-smokers (65.4%), and in subjective good health (48.0%) were high. The results showed that 70.6% of the subjects were concerned about infection due to COVID-19, 45% were worried about death due to the COVID-19 pandemic, 78.6% were concerned about economic damage due to the COVID-19 pandemic, and 20.1% were dissatisfied with life due to the COVID-19 pandemic.

### 3.2. Characteristics of Subjects Who Were Life Dissatisfied: Potential Factors

Table 1 shows the general characteristics of subjects according to life dissatisfaction. The results of chi-square tests showed that residential area type, age, gender, education level, mean monthly household income, smoking, binge-drinking, subjective health level, concerns about COVID-19 infection, fear of death due to COVID-19 infection, concerns about criticism from others due to COVID-19 infection, concerns about family’s COVID-19 infection (e.g., older adults and children), concerns about economic damage (e.g., unemployment) due to COVID-19, changes in sleeping hours after the COVID-19 pandemic, and number of meetings with friends or neighbors after the outbreak of COVID-19 were significantly different for the life dissatisfied groups compared with the life satisfied groups (*p* < 0.05).

### 3.3. Development of Bayesian Nomogram for Predicting the Subjective Life Dissatisfaction of Korean Adults in COVID-19 Pandemic

The Bayesian classification model is presented in Table 2.The analysis results of the crude model, which identified the individual influence factors of life dissatisfaction due to the COVID-19 pandemic as age, gender, residential area, education level, mean monthly household income, smoking, subjective health, concerns about infection due to the COVID-19 pandemic, fear of death due to the COVID-19 pandemic, concerns about criticism from others due to COVID-19 infection, concerns about infection of the weak due to the COVID-19 pandemic, concerns about economic damage due to the COVID-19 pandemic, changes in the number of meetings after the outbreak of the COVID-19 pandemic, and changes in sleeping hours after the COVID-19 pandemic, showed that these factors were significantly associated with depression in older adults living alone (*p* < 0.05). The analysis results of the adjusted model, which included all variables to identify multiple risk factors for life dissatisfaction due to the COVID-19 pandemic, confirmed the risk factors and protective factors regarding life dissatisfaction (*p* < 0.05). Age 50 or above was a protective factor against life dissatisfaction due to the COVID-19 pandemic. People between 50 and 59 years old and those 60 years old or older had a 10% (OR = 0.90, 95% CI: 0.82~0.99) and 12% (OR = 0.88, 95% CI: 0.80~0.96) lower life dissatisfaction risk, respectively, compared to those between 19 and 29 years old (*p* < 0.05). Residential area was also a protective factor. Compared to adults living in cities, adults living in rural areas had a 19% lower risk of life dissatisfaction due to the COVID-19 pandemic (OR = 0.81, 95% CI: 0.79~0.83) (*p* < 0.05). Moreover, adults with increased sleeping hours (OR = 0.86, 95% CI: 0.82~0.91) and adults with similar sleeping hours (OR = 0.49, 95% CI: 0.47~0.51) had a lower risk of life dissatisfaction due to the COVID-19 pandemic than adults with decreased sleeping hours after the COVID-19 pandemic (*p* < 0.05).

The confirmed independent risk factors for life dissatisfaction due to the COVID-19 pandemic were being 30–39 years old (OR = 1.23, 95% CI: 1.12, 1.35), being female (OR = 1.38, 95% CI = 1.32, 1.43), having middle school graduation education level (OR = 1.25, 95% CI = 1.19, 1.31), having high school graduation education level (OR = 1.37, 95% CI = 1.30, 1.43), having college graduation education level or above (OR = 1.44, 95% CI = 1.36, 1.52), having a mean monthly household income between KRW 1 and 2.99 million (OR = 1.11, 95% CI = 1.06, 1.15), having poor subjective health (OR = 1.18, 95% CI = 1.13, 1.23), having concerns about infection due to the COVID-19 pandemic (OR = 1.32, 95% CI = 1.24, 1.41), having concerns about economic damage due to the COVID-19 pandemic (OR = 1.31, 95% CI = 1.23, 1.38), and experiencing a change in the number of meetings due to the COVID-19 pandemic. (*p* < 0.05).

**Figure 2:**
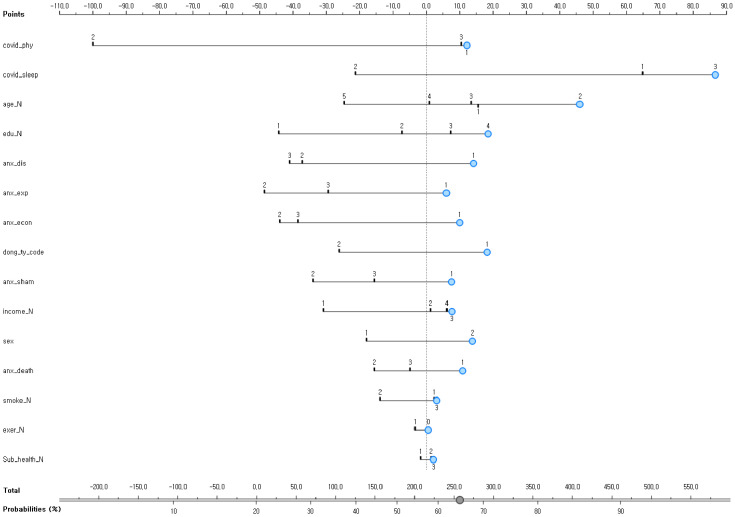
A Bayesian nomogram for predicting the subjective life dissatisfaction of Korean adults in COVID-19 pandemic in Korea. covid_phy = number of meetings with friends or neighbors after the outbreak of COVID-19 (1 increased, 2 similar, or 3 decreased); covid_sleep = changes in sleeping hours after the COVID-19 pandemic (1 increased, 2 similar, or 3 decreased); age_N = (1 = 19–29, 2 = 30–39, 3 = 40–49, 4 = 50–59, 5 = 60+years); edu_N = education level (1 elementary school graduation or below, 2 middle school graduation, 3 high school graduation, 4 college graduation or above); anx_dis = concerns about COVID-19 infection (1 concerned, 2 indifferent, 3 not concerned); anx_econ = concerns about economic damage due to COVID-19 (1 concerned, 2 indifferent, 3 not concerned); dong_by_code = residential area type (1 urban, 2 rural); anx_sham = concerns about criticism from others due to COVID-19 infection (1 concerned, 2 indifferent, 3 not concerned); income_N = mean monthly household income (1 = less than KRW 1 million, 2 = KRW 1 to 3 million, 3 = KRW 3 to 5 million, 4 = KRW 5 million or more); sex = (1 male, 2 female); anx_death = fear of death due to COVID-19 infection (1 concerned, 2 indifferent, 3 not concerned); smoke_N = smoking (1 current smoker, 2 past smoker, 3 non-smoker); Sub_health_N = subjective health level (1 good, 2 average, 3 bad).

Figure 2 shows the Bayesian nomogram for predicting the subjective life dissatisfaction of Korean adults in the COVID-19 pandemic in Korea. Changes in the number of meetings after the outbreak of the COVID-19 pandemic showed the highest influence among the risk factors for predicting the subjective life dissatisfaction of Korean adults in the COVID-19 pandemic. For example, the nomogram developed in this study showed that “females who were between 30 and 39 years old, living in urban areas, with fewer meetings and fewer sleeping hours, concerned about infection for themselves and for the weak in the family due to the COVID-19 pandemic, concerned about death, with a mean household monthly income of KRW 3–5 million, who were non-smokers, with poor subjective health, and with an education level of college graduation or above” had a 66% chance of life dissatisfaction due to the COVID-19 pandemic (Figure 2).

**Figure 3:**
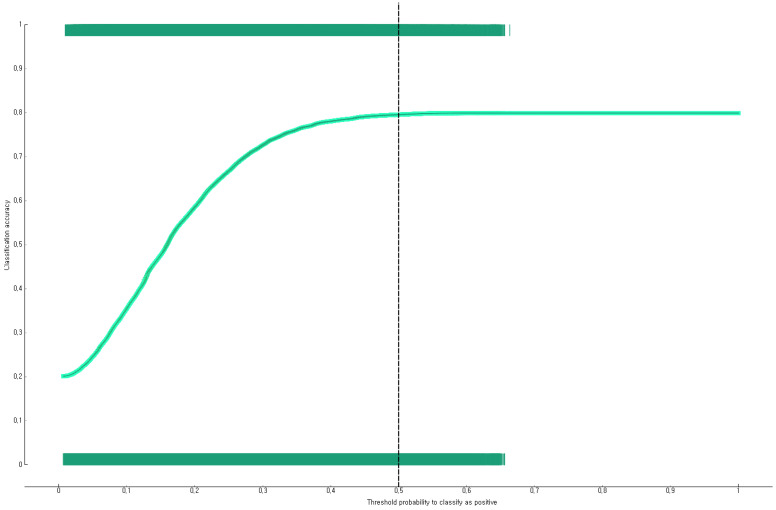
General accuracy (10-fold validation) of Bayesian nomogram for predicting the subjective life dissatisfaction of Korean adults in COVID-19 pandemic in Korea.

**Figure 4:**
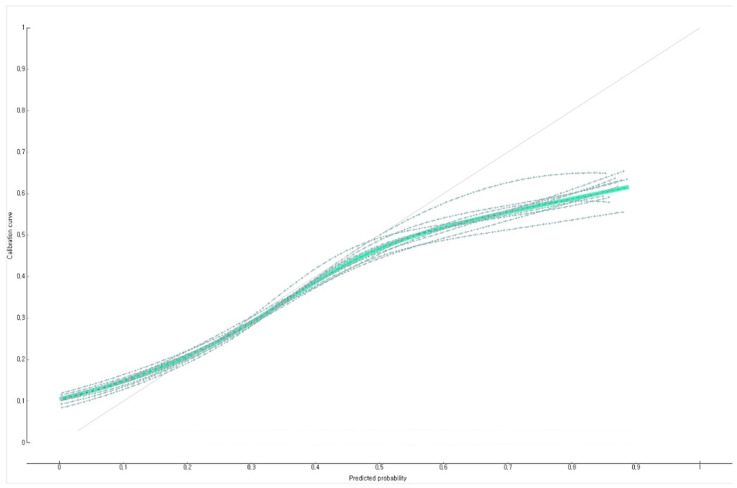
Calibration plot (10-fold validation) of Bayesian nomogram for predicting the subjective life dissatisfaction of Korean adults in COVID-19 pandemic in Korea.

The prediction performance of the developed Bayesian nomogram was validated using the F1-score, general accuracy, precision, recall, and a calibration plot. The results of a 10-fold cross-validation showed that the F1-score, general accuracy (Figure 3), precision, and recall values of the Bayesian nomogram developed in this study were 0.73, 0.80, 0.73, and 0.80, respectively. This study compared the prediction probability and observation probability of a group of Korean adults with subjective life dissatisfaction in the COVID-19 pandemic with a group of Korean adults with subjective life satisfaction in the COVID-19 pandemic using a calibration plot (Figure 4) and the chi-square test, and found that there was no significant difference between them (*p* = 0.150).

## 4. Discussion

Identifying the life satisfaction of populations and high-risk groups is important in terms of the well-being of people and society in an unpredictable disaster situation such as the COVID-19 pandemic [16]. Although the South Korean government’s regulations have succeeded in blocking the COVID-19 pandemic, there are few studies on how much it has affected adults living in the community.

Our findings show that the COVID-19 pandemic did negatively affect people’s life satisfaction, with this relationship depending on the individual’s age, gender, area of living, education level, mean monthly household income, subjective health, concern about infection/death/criticism of others/economic damage due to the COVID-19 pandemic, changes in the number of meetings, and changes in sleeping hours. Multiple studies [17,18,19,20] have shown that people’s life satisfaction and happiness decreased due to the COVID-19 pandemic. Cheng et al. (2020) [17] analyzed Google Trends data and reported that the COVID-19 pandemic increased the search intensity for boredom, loneliness, worry, and sadness, which are words related to life satisfaction, by a great deal in Europe and the United States. Bidzan-Bluma et al. (2020) [21] also reported that the quality of life and the life satisfaction of adults in Germany and Poland decreased after the COVID-19 pandemic.

In particular, Bidzan-Bluma et al. (2020) [21] confirmed that the quality of life, life satisfaction, and sense of well-being of middle-aged and young people were lower than those of older adults due to the COVID-19 pandemic, which agrees with the results of this study. In a study similar to the current study, Lee et al. (2020) [22] examined the change in the quality of life before and after the COVID-19 pandemic in South Korean communities (for people 18 years old and older) and showed that it was different for different age groups. The quality of life decreased drastically in the middle-aged group (31–50 years) [22]. Previous studies [21,22] argued that the change in life satisfaction due to the COVID-19 pandemic was relatively larger in the young compared to the old because the old had experienced various disasters during their lifetimes and they had become more resilient. Therefore, it is necessary to provide customized support according to age, to reduce people’s dissatisfaction with life after the COVID-19 pandemic, based on the results of this study. In particular, it is necessary to systematize support for young people and middle-aged people and offer training on responding to disasters such as infectious diseases.

The results of this study confirmed that the change in the number of meetings after the COVID-19 pandemic was a major risk factor for life dissatisfaction. One of the important reasons for this result is the restricted social participation as a result of social distancing, due to the government’s intensive quarantine measures after the COVID-19 pandemic [23,24,25]. In other words, it is highly likely that social distancing has decreased the life satisfaction of modern people who value social activities and social relationships [26,27]. Previous studies [28,29] showed that social encounters with friends and neighbors are related to subjective life satisfaction, which supports this possibility.

The South Korean government implemented intensive social distancing to prevent the spread of COVID-19 [30]. For example, elementary, middle, high school, and college classes were switched to online classes, telecommuting was implemented widely in the workplace, and many older adults were isolated at home because the access to welfare centers and senior-citizen centers was restricted [30]. In addition, after the COVID-19 pandemic, the self-employed in the face-to-face service industry suffered from large operating losses due to the reduction in business hours and control of the number of visitors, and the rate of household debt also increased rapidly [31]. Various changes in lifestyle, such as fear due to COVID-19 infection, social relationship dissolution, economic changes, and changes in health due to reduced physical activities are highly likely to reduce people’s life satisfaction. In fact, many empirical studies have shown that life satisfaction decreased during the COVID-19 epidemic [21,22,32].

This study confirmed that concern about COVID-19 was also a major risk factor for life dissatisfaction. Duong et al. (2021) [18] conducted an online survey of college students in Vietnam and reported that fear and anxiety in young people about COVID-19 were strongly and negatively associated with life satisfaction, which was similar to the results of this study. In addition, Satici et al. (2020) [33] surveyed 1304 adults in a Turkish community and revealed that the fear of COVID-19 was negatively related to life satisfaction. Therefore, the government or local communities should offer effective training related to infectious diseases or operate customized psychological counseling support programs to prevent or minimize psychological difficulties such as anxiety, in addition to offering economic support to increase the life satisfaction of people after the COVID-19 pandemic.

Another finding of this study involved identifying multiple risk factors for life dissatisfaction of communities using the nomogram. In this study, “females who were between 30 and 39 years old, living in urban areas, with fewer meetings and sleeping hours, concerned about infection for themselves and the weak in the family due to the COVID-19 pandemic, concerned about death, with a mean household monthly income of KRW 3–5 million, who were non-smokers, with poor subjective health, and an education level of college graduation or above” were predicted to have a high risk (66%) of life dissatisfaction due to the COVID-19 pandemic. Therefore, continuous support is needed for diagnosing the life satisfaction of those with these multiple risk factors and providing continuous support from a social aspect.

Previous studies [7,8,9] that explored the influencing factors for life satisfaction mainly used regression analysis to identify individual risk factors for life satisfaction. Only a few studies examined life satisfaction and multiple risk factors for community populations after the outbreak of COVID-19. Therefore, the results of previous studies cannot be directly compared with the results of this study. The Korea Institute for Health and Social Affairs (2021) [34] analyzed the damage done by the COVID-19 pandemic by class and reported the statistics that specific groups (e.g., the self-employed, middle- and low-income groups, men in their 40s and 50s, and women in their 30s) showed a larger decrease in life satisfaction and a larger increase in depression. The results were similar to the results of this study. Oh (2020) [35] statistically examined the changes in the labor market after the COVID-19 pandemic and also confirmed that overall life satisfaction generally decreased in the 20–34 age group, living in the Seoul metropolitan area, and in the female group. Therefore, more social attention and countermeasures are needed to enhance life satisfaction for groups at high risk of life dissatisfaction after COVID-19, as found in this study. Furthermore, more studies are needed to explore the multiple risk factors associated with life satisfaction.

During the COVID-19 pandemic, many people refrained from contact with people as much as possible, spent more time at home, and reduced their level of physical activity to avoid infection [36]. In many cases, people lost their normal daily life, had difficulties in performing important tasks and implementing plans, and were isolated from the outside world [32]. Moreover, people were worried because they could not predict their future [22]. In other words, the COVID-19 pandemic changed people’s lives and caused a great deal of disruption. This is a common phenomenon occurring all over the world, not limited to a few specific countries. In most countries, many social activities are restricted to non-face-to-face only to prevent the spread of COVID-19, and individuals’ daily activities are also restricted or prohibited when not essential [37]. Even though quarantine measures such as social distancing have been implemented for the purpose of protecting the lives and safety of individuals and countries from the infectious disease [37], interest in, and alternatives for, handling the problems caused by it are still insufficient. Therefore, effective policy intervention in terms of social psychology, as well as economic support, is required to minimize the damage caused by disasters and increase life satisfaction for the groups at high risk of life dissatisfaction after COVID-19, based on the results of this study. Furthermore, the study suggests the need to systematically provide disaster response information and education for disaster situations such as infectious diseases, for groups at high risk of life dissatisfaction.

The Bayesian nomogram developed for predicting life dissatisfaction after the COVID-19 pandemic can show the importance of predictors at a glance and indicate the probability of occurrence intuitively, which are advantages. The limitations of this study are as follows. First, in this study, the life satisfaction of local populations was surveyed only with a self-report questionnaire. When only self-report questionnaires are analyzed, recall bias may occur. Therefore, future studies are needed to minimize recall bias by combining qualitative and quantitative studies with the self-report survey. Second, other potential variables related to life satisfaction may exist in addition to the variables included in the predictive model of this study. Follow-up studies are required to develop models that can predict life satisfaction by including more variables such as clinical data and pattern-of-life data such as log data. Third, since it is a cross-sectional study, the results of this study cannot be interpreted as causal relationships, even though risk factors for life satisfaction are found. A prospective cohort study is required to understand the causality of life satisfaction and the risk factors identified in this study.

## 5. Conclusions

The results of this study suggest that the government needs not only to provide economic support but also to support education on infectious diseases and customized psychological counseling programs for those at high risk of life dissatisfaction after the COVID-19 pandemic. Furthermore, based on the results of this study, it is necessary to pay attention to “females between 30 and 39 years old, living in urban areas, with fewer meetings and sleeping hours, concerned about infection for themselves and the weak in the family due to the COVID-19 pandemic, concerned about death, with a mean household monthly income of KRW 3–5 million, who are non-smokers, with poor subjective health, and an education level of college graduation or above”, in order to increase the life satisfaction of adults living in the community during the COVID-19 pandemic.

## Figures and Tables

**Table 1:** The characteristics of the life dissatisfied subjects during the COVID-19 pandemic, *n* (%).

Variables	Life Dissatisfied		*p*
	Yes (*n* = 45,807)	No (*n* = 182,001)	
Age			<0.001
19–29	1045 (22.1)	2681 (77.9)	
30–39	6673 (26.5)	18,555 (73.5)	
40–49	7829 (21.8)	28,016 (78.2)	
50–59	8987 (20.2)	35,464 (79.8)	
60+	16,558 (17.2)	79,618 (82.8)	
Gender			<0.001
Male	18,573 (18.0)	84,818 (82.0)	
Female	27,234 (21.9)	97,183 (78.1)	
Residential area type			<0.001
Urban	28,874 (22.5)	99,621 (77.5)	
Rural	16,933 (17.1)	82,380 (82.9)	
Education level			<0.001
Elementary school graduation or below	7533 (15.2)	42,174 (84.8)	
Middle school graduation	4895 (19.2)	20,582 (80.8)	
High school graduation	13,943 (21.0)	52,375 (79.0)	
College graduation or above	19,370 (22.5)	66,678 (77.5)	
Mean monthly household income			<0.001
Less than KRW 1 million	5095 (16.5)	25,731 (83.5)	
KRW 1 to 3 million	11,699 (20.3)	46,032 (79.7)	
KRW 3 to 5 million	9390 (21.1)	35,164 (78.9)	
KRW 5 million or more	10,107 (20.9)	38,285 (79.1)	
Smoking			<0.001
Current smoker	7599 (20.4)	29,654 (79.6)	
Past smoker	7683 (18.4)	33,983 (81.6)	
Non-smoker	30,518 (20.5)	118,346 (79.5)	
Regular exercise			0.050
No	40,069 (20.2)	158,517 (79.8)	
Yes	5725 (19.7)	23,359 (80.3)	
Subjective health level			0.043
Good	21,764 (19.9)	87,656 (80.1)	
Average	17,837 (20.3)	70,092 (79.7)	
Bad	6204 (20.4)	24,248 (79.6)	
Concerns about COVID-19 infection			<0.001
Concerned	35,262 (21.9)	125,480 (78.1)	
Indifferent	7035 (15.9)	37,306 (84.1)	
Not concerned	3508 (15.5)	19,170 (84.5)	
Fear of death due to COVID-19 infection			<0.001
Concerned	22,008 (21.5)	80,358 (78.5)	
Indifferent	9685 (18.2)	43,417 (81.8)	
Not concerned	14,081 (19.5)	58,091 (80.5)	
Concerns about criticism from others due to COVID-19 infection			<0.001
Concerned	36,300 (21.1)	136,073 (78.9)	
Indifferent	4844 (16.2)	25,041 (83.8)	
Not concerned	4619 (18.2)	20,707 (81.8)	
Concerns about family’s COVID-19 infection (e.g., older adults and children)			<0.001
Concerned	37,916 (20.9)	143,781 (79.1)	
Indifferent	2603 (14.7)	15,081 (85.3)	
Not concerned	1872 (16.7)	9350 (83.3)	
Concerns about economic damage (e.g., unemployment) due to COVID-19			<0.001
Concerned	38,271 (21.4)	140,703 (78.6)	
Indifferent	4004 (15.2)	22,364 (84.8)	
Not concerned	3519 (15.7)	18,854 (84.3)	
Number of meetings with friends or neighbors after the outbreak of COVID-19			<0.001
Increased	152 (21.6)	552 (78.4)	
Similar	2874 (10.4)	24,823 (89.6)	
Decreased	40,049 (21.4)	146,701 (78.6)	
Changes in sleeping hours after the COVID-19 pandemic			<0.001
Increased	6864 (29.4)	16,484 (70.6)	
Similar	32,533 (17.6)	152,514 (82.4)	
Decreased	6403 (33.0)	12,993 (67.0)	

**Table 2:** Prediction of dissatisfaction with life due to the COVID-19 pandemic. Figures in parentheses are OR—odds ratio, CI— 95% confidence interval.

Variables	Crude Model	*p*	Adjusted Model	*p*
Age				
19–29 (ref.)	1.00		1.00	
30–39	1.27 (1.17, 1.36)	<0.001	1.23 (1.12, 1.35)	<0.001
40–49	0.98 (0.91, 1.06)	0.672	0.97 (0.89, 1.06)	0.599
50–59	0.89 (0.83, 0.96)	0.002	0.90 (0.82, 0.99)	0.033
60+	0.73 (0.68, 0.78)	<0.001	0.88 (0.80, 0.96)	0.007
Gender				
Male (ref.)	1.00		1.00	
Female	1.28 (1.25, 1.31)	<0.001	1.38 (1.32, 1.43)	<0.001
Residential area type				
Urban (ref.)	1.00		1.00	
Rural	0.70 (0.69, 0.72)	<0.001	0.81 (0.79, 0.83)	<0.001
Education level				
Elementary school graduation or below (ref.)	1.00		1.00	
Middle school graduation	1.33 (1.28, 1.38)	<0.001	1.25 (1.19, 1.31)	<0.001
High school graduation	1.49 (1.44, 1.53)	<0.001	1.37 (1.30, 1.43)	<0.001
College graduation or above	1.62 (1.57, 1.67)	<0.001	1.44 (1.36, 1.52)	<0.001
Mean monthly household income				
Less than KRW 1 million	0.75 (0.72, 0.77)	<0.001	1.02 (0.97, 1.08)	0.314
KRW 1 to 3 million	0.96 (0.93, 0.99)	0.013	1.11 (1.06, 1.15)	<0.001
KRW 3 to 5 million	1.01 (0.98, 1.04)	0.478	1.01 (0.97, 1.04)	0.720
KRW 5 million or more (ref.)	1.00		1.00	
Smoking				
Current smoker (ref.)	1.00		1.00	
Past smoker	0.88 (0.85, 0.91)	<.001	0.92 (0.88, 0.97)	0.001
Non-smoker	1.00 (0.97, 1.03)	0.662	0.81 (0.78, 0.85)	<0.001
Subjective health level				
Good (ref.)	1.00		1.00	
Average	1.02 (1.00, 1.05)	0.029	1.03 (1.00, 1.06)	0.019
Bad	1.03 (0.99, 1.06)	0.063	1.18 (1.13, 1.23)	<0.001
Concerns about COVID-19 infection				
Concerned	1.53 (1.47, 1.59)	<0.001	1.32 (1.24, 1.41)	<0.001
Indifferent	1.03 (0.98, 1.07)	0.182	1.01 (0.94, 1.07)	0.755
Not concerned (ref.)	1.00		1.00	
Fear of death due to COVID-19 infection				
Concerned	1.13 (1.10, 1.15)	<0.001	0.98 (0.94, 1.02)	0.290
Indifferent	0.92 (0.89, 0.94)	<0.001	0.88 (0.85, 0.92)	<0.001
Not concerned (ref.)	1.00		1.00	
Concerns about criticism from others due to COVID-19 infection				
Concerned	1.19 (1.15, 1.23)	<0.001	1.02 (0.97, 1.07)	0.396
Indifferent	0.86 (0.83, 0.90)	<0.001	0.91 (0.85, 0.97)	0.006
Not concerned (ref.)	1.00		1.00	
Concerns about family’s COVID-19 infection (e.g., older adults and children)				
Concerned	1.31 (1.25, 1.38)	<0.001	1.04 (0.96, 1.12)	0.276
Indifferent	0.86 (0.80, 0.92)	<0.001	0.93 (0.84, 1.01)	0.109
Not concerned (ref.)	1.00		1.00	
Concerns about economic damage (e.g., unemployment) due to COVID-19				
Concerned	1.45 (1.40, 1.51)	<0.001	1.31 (1.23, 1.38)	<0.001
Indifferent	0.96 (0.91, 1.01)	0.098	1.05 (0.97, 1.12)	0.183
Not concerned (ref.)	1.00		1.00	
Number of meetings with friends or neighbors after the outbreak of COVID-19				
Increased	1.01 (0.84, 1.20)	0.925	1.06 (0.85, 1.34)	0.575
Similar	0.42 (0.40, 0.44)	<0.001	0.52 (0.49, 0.54)	<0.001
Decreased (ref.)	1.00		1.00	
Changes in sleeping hours after the COVID-19 pandemic				
Increased	0.84 (0.81, 0.88)	<0.001	0.86 (0.82, 0.91)	<0.001
Similar	0.43 (0.42, 0.44)	<0.001	0.49 (0.47, 0.51)	<0.001
Decreased (ref.)	1.00		1.00	
